# Spontaneous Intraperitoneal Rupture of a Hepatic Hydatid Cyst with Subsequent Anaphylaxis: A Case Report

**DOI:** 10.1155/2013/320418

**Published:** 2013-03-06

**Authors:** Benjamin Tinsley, Aula Abbara, Raghunandan Kadaba, Hemant Sheth, Gurjinder Sandhu

**Affiliations:** ^1^Department of Upper Gastrointestinal Surgery, Ealing Hospital NHS Trust, London UB1 3HW, UK; ^2^Department of Infectious Disease, Ealing Hospital NHS Trust, London UB1 3HW, UK

## Abstract

Hydatid cyst rupture into the abdomen is a serious complication of cystic hydatid disease of the liver (Cystic Echinococcosis) with an incidence of up to 16% in some series and can result in anaphylaxis or anaphylactoid reactions in up to 12.5% of cases. At presentation, 36–40% of hydatid cysts have ruptured or become secondarily infected. Rupture can be microscopic or macroscopic and can be fatal without surgery. Hydatid disease of the liver is primarily caused by the tapeworm *Echinococcus granulosus* and occurs worldwide, with incidence of up to 200 per 100,000 in endemic areas. Our case describes a 24-year-old Bulgarian woman presenting with epigastric pain and evidence of anaphylaxis. Abdominal CT demonstrated a ruptured hydatid cyst in the left lobe of the liver. A partial left lobe hepatectomy, cholecystectomy, and peritoneal washout was performed with good effect. She was treated for anaphylaxis and received antihelminthic treatment with Albendazole and Praziquantel. She made a good recovery following surgery and medical treatment and was well on follow-up. Intraperitoneal rupture with anaphylaxis is a rare occurrence, and there do not seem to be any reported cases from UK centres prior to this.

## 1. Introduction

Hydatid disease (Echinococcosis) of the liver is a parasitic infection primarily caused by the larvae of the cestode *Echinococcus granulosus*. This tapeworm is responsible for cystic hydatid disease (Cystic Echinococcosis), which is the most common form [1]. Worldwide incidence is between 1 and 200 per 100,000 [1]. Endemic regions include the Mediterranean littoral (especially Turkey, Greece, Cyprus, and Spain), central Russia, China, and Australasia [1–5]. In Bulgaria—our patient's country of origin—the annual incidence in children increased from 0.7 per 100,000 in 1971–82 to 5.4 in 1995 [1, 4].

The adult cestode develops in the small intestine of the definitive host (most commonly dogs) and releases eggs which are dispersed in the stools of the host [2]. When eggs are ingested by an intermediate host (usually sheep or cattle), embryos are released into the duodenum and pass into the portal and lymphatic systems [2]. Humans act as “aberrant” intermediate hosts by accidentally ingesting eggs via infected vegetables or water, or touching dogs with contaminated hair [1, 3]. The most common site for cystic disease is the liver (around 75% [1]); lung (up to 25% [5]) is the second most common site. Less frequently cysts develop in the spleen, kidneys, heart, bones, and central nervous system [4].

The embryo of *Echinococcus granulosus* develops into a parent cyst which is formed by two membranous layers: an inner germinative membrane and an outer hyaline membrane. A third peripheral layer of fibroblasts is derived from the host tissue; this develops into the pericyst [3]. Brood capsules containing numerous protoscolices (which will develop into the head of the adult cestode) develop by budding of the germinative membrane [1]. Daughter cysts are often seen within the parent cyst [1].

Cysts may be located in any area of the liver and may be solitary or multiple. The right lobe is involved in 75% of cases [2], and 20–40% of patients have multiple cysts [1, 4]. The rate of cyst growth is variable, ranging from 1 to 5 cm in diameter per year [4], and cysts are rarely symptomatic until they reach 10 cm in diameter [2]. Cysts can attain a volume of several litres and contain many thousands of protoscolices [4]. Estimates of rupture rates vary with different series but are reported in up to 16% [6]; risk of rupture is associated with young age, superficial localisation, trauma, and large size [5]. Rupture can result in complications including abdominal pain, anaphylaxis, and death.

## 2. Case

A 24-year-old Bulgarian woman presented to the Emergency Department with a 2-hour history of acute onset epigastric pain which had migrated to the lower abdomen. She reported mild, intermittent, dull epigastric pain for the preceding year. There was no history of trauma or any past medical history of note. At presentation, she was haemodynamically stable with a pulse rate of 69 bpm and blood pressure of 108/36 mmHg. There was no respiratory compromise and O_2_ saturation was 98% on room air. Her temperature was 36.9°C. Palpation of the abdomen elicited significant epigastric and suprapubic tenderness with guarding and percussion tenderness. A faint erythematous rash was noted over her face and arms. Thirty minutes later she became shocked, tachypnoeic, and hypothermic at 35.2°C. The rash was more extensive, appeared urticarial and she had developed periorbital oedema. Her condition was stabilised with aggressive fluid resuscitation and intravenous steroids (Hydrocortisone 200 mg) and antihistamines (Chlorpheniramine 20 mg).

**Figure 1:**
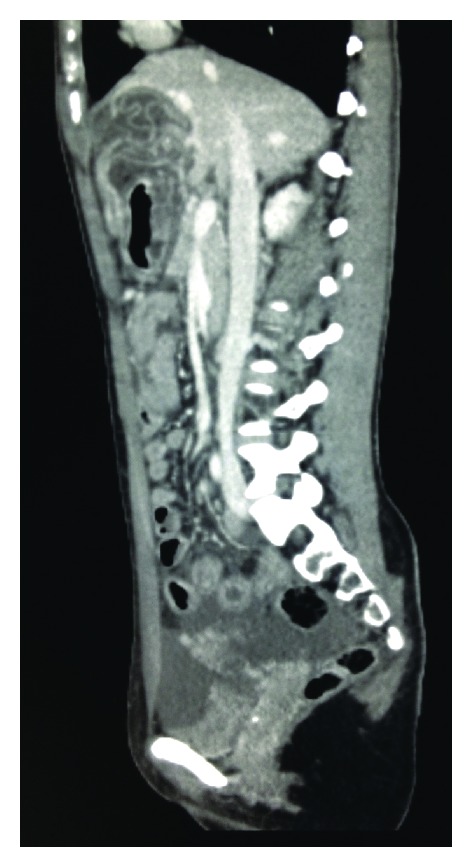
Contrast CT (Sagittal section) demonstrating the ruptured hydatid cyst within the anterior portion of the left lobe of the liver, with serpiginous strands representing the collapsed membrane. Significant free fluid is noted within the pelvis.

Initial blood investigations were normal apart from a haemoglobin of 16.1 g/dL (12–16), glucose of 11.7 mmol/L (3–7), and lactate of 2.73 mmol/L (0.5–2.2). Liver and renal function tests were normal and there was no leucocytosis or eosinophilia. Venous blood gas demonstrated a pH of 7.287 (7.35–7.45), bicarbonate of 22.6 mEq/L (22–29), and base excess of −1.6 (−2 to 2) with normal pO_2_ and pCO_2_. Urine dipstick and *β*-HCG were negative. Contrast CT of her abdomen and pelvis revealed a large, well-defined area of low density occupying almost the entire left lobe of the liver with serpiginous enhancing strands within. Significant free fluid was noted throughout the abdomen and pelvis (see Figure 1). The appearances were highly suggestive of a ruptured hepatic hydatid cyst.

**Figure 2:**
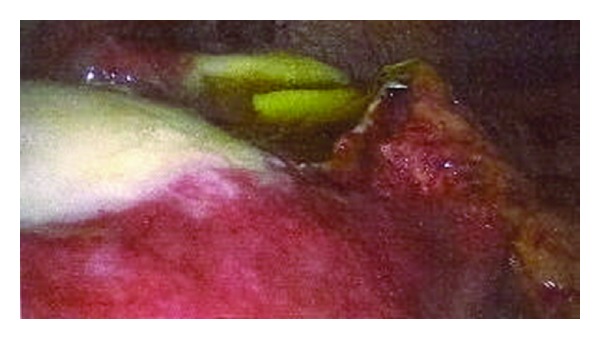
Intra-operative laparoscopy image demonstrating the ruptured cyst and folded hyaline membrane within. Hydatid fluid is leaking from the ruptured cyst.

The decision was made to perform a diagnostic laparoscopy with a view to proceeding to laparotomy if required. Laparoscopy revealed a large ruptured cystic mass within the left lateral lobe of the liver, almost completely replacing the hepatic parenchyma (see Figure 2). There were also features of cholecystitis noted. Large amounts of free fluid were noted in all four quadrants. Removal of the cyst was achieved by way of an open left partial hepatectomy and the peritoneal cavity was washed out with hypertonic saline with a drain left in the subhepatic space. A cholecystectomy was also performed. Post-operatively she was managed in ITU and was commenced on Albendazole and Tazocin (Piperacillin/Tazobactam) with ongoing therapy for anaphylaxis. Praziquantel was also commenced but the patient subsequently became bradycardic to 30 bpm (although remained haemodynamically stable). No other cause was identified and resolved after discontinuation of Praziquantel. She was discharged 9 days post-operatively on reducing dose oral steroids and 3 cycles of Albendazole (4 weeks on, 2 weeks off). At follow-up, she remained well.

**Figure 3:**
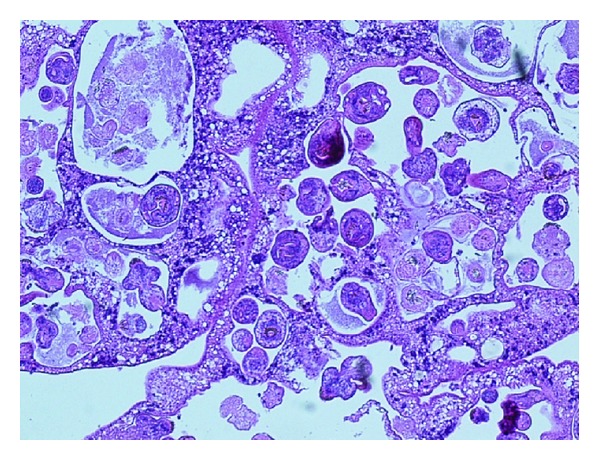
H&E stain of hydatid cyst contents demonstrating numerous scolices (with thanks to Dr. Faris Kubba, Consultant Histopathologist, Ealing Hospital NHS Trust).

Histology of the resected tissue confirmed the presence of a hyalinised, PAS-positive pseudocapsule (Brodie's capsule) with numerous scolices present, thus confirming the diagnosis of a hydatid cyst (see Figure 3). Histology of the gall bladder showed mild cholecystitis. No protoscolices or hooklets were seen in the drain fluid. Echinococcal serology (Hydatid ELISA) was positive.

## 3. Discussion

Hepatic hydatid cysts may remain clinically silent for many years and are often an incidental finding on ultrasound performed for another reason [2]. The diagnosis is often made when complications occur; usually jaundice but may also include rupture or secondary infection [2]. At the time of diagnosis 36–40% of cysts have ruptured or become secondarily infected [2]. A mass is rarely palpated before the cyst reaches 20 cm in diameter [2] with cysts in the anterior area of the liver more readily palpated. Cysts close to the peritoneal cavity may rupture into the free peritoneum, or less commonly the gastrointestinal tract or right renal pelvis [2]. Rupture may lead to development of extrahepatic cysts and may also cause symptoms of an allergic origin such as marked eosinophilia, urticaria or anaphylaxis. More commonly, rupture occurs into bile ducts resulting in biliary obstruction, cholangitis, or biliary colic [2]. Marked elevations of bilirubin and alkaline phosphatase occur in these cases [7]. Cysts may also erode through the diaphragm and rupture into the pleural or pericardial cavities, lung, or bronchi [2]. Cyst suppuration has been reported in 5–40% of patients [1]. Glomerular deposits of hydatid antigen may result in membranous glomerulonephritis [3].

The key tools for diagnosis are imaging and serology. Abdominal ultrasound is considered the gold standard [1] as it is non-invasive, inexpensive and can visualise the cysts immediately. MRI is preferable to CT, particularly for pre-surgical evaluation [1] as it gives better visualisation of liquid areas within the cyst. Several serological tests are available, but IgG ELISA to the antigen B-rich fraction is the most sensitive (>95%) and specific [7]. Hepatic cysts are more likely to elicit an immune response [4]. Eosinophilia of greater than 7% is found in 30% of patients [3].

Treatment aims to eliminate the parasite and to prevent recurrence, in order to minimise morbidity and mortality risk [1]. Medical treatment is with the benzimidazole Albendazole, but cannot be regarded as definitive treatment alone [3]. Albendazole has better absorption than other benzimidazoles but many months of therapy are still required, occasionally in conjunction with Praziquantel [7]. Generally, 30% of cysts disappear, 30–50% degenerate, and 20–40% remain unchanged [3, 4].

Surgical management of unruptured cysts ranges from radical (such as pericystectomy or liver resection) to conservative (de-roofing, marsupialisation or capitonnage) using open or laparoscopic techniques [1, 4, 5]. Radical techniques have a lower recurrence rate but higher operative risk. Overall recurrence rates vary from 2% to 25%, and operative mortality ranges from 0.5% to 4% [1, 4]. Partial hepatectomy is advocated for cysts involving the left lateral segment, with a recurrence rate of 8–22% [2]. Adjunctive benzimidazoles are used to reduce the risk of anaphylaxis and dissemination. Surgical removal of all cysts with associated chemotherapy should be the ideal therapeutic option; however, the level of evidence is low [6, 8]. More recently, percutaneous techniques such as PAIR (“Puncture-Aspiration-Injection-Re-aspiration”) have been developed, which may be comparable or even superior to surgery [9].

The most frequent sites of rupture are the biliary tract (12%) and thorax (2.2%) [8]. Rare cases have involved intrabiliary and intraperitoneal rupture simultaneously [10]. Intraperitoneal rupture is a rare but serious complication of hepatic Cystic Echinococcosis and presents a unique challenge to the surgeon. Intraperitoneal rupture was reported in 1.6% of patients with Cystic Echinococcosis of the liver in a large multicentre retrospective study [8]; however, other citations place this figure as high as 16% [6]. In Turkey—the most frequent source of case reports involving intraperitoneal rupture—the rate has been noted at 7.8% [5, 11]. Risk factors include young age, a cyst diameter of more than 10 cm, and superficial cyst location [5]. Rupture of cysts is unpredictable and can occur spontaneously [10–12], as a result of trauma [5, 6, 13, 14] or iatrogenically. Trauma is the most frequent cause [5]. The usual presentation is that of an acute abdomen requiring emergency surgery to remove the intraperitoneal fluid and eradicate the cysts [6, 8, 10, 11]. However, rupture can cause only minimal symptoms or can even be clinically silent, presenting years later with disseminated intra-abdominal disease [8]. After intraperitoneal rupture of liver cysts, mortality rates of 0–23.5% have been reported [11, 15]. Morbidity ranges from 20% to 35.3% [8, 15], with cited recurrence rates between 6.7% and 28.6% of cases [6, 11]. Currently, no clear guidelines are available for management of intraperitoneal rupture of cysts [5]. Intraperitoneal rupture of a cyst is difficult to cure due to the remaining protoscolices forming extrahepatic daughter cysts. Recurrence is almost inevitable, and benzimidazoles are essential in these cases [2]. Cholangitis is usually treated with biliary drainage techniques [3].

Ruptured cysts fall into three categories: contained, communicating, and direct [16]. Contained rupture involves only the endocyst and the cyst contents remain within the confines of the pericyst. Communicating rupture involves tear of the endocyst with escape of contents via biliary radicules (or bronchioles) that have been incorporated into the pericyst. Tear of the pericyst and endocyst allowing spillage of contents into the peritoneal or pleural cavity is direct rupture [13].

Anaphylaxis is a rare (1–12.5% [5, 6]) but recognised sequela of cyst rupture and can occur after all types of rupture. In one series, anaphylaxis complicated 10% of all intraperitoneal ruptures [1]. Less severe allergic reactions can occur in up to 25% of patients with intraperitoneal rupture of cysts [6]. Numerous cases of intraperitoneal rupture with anaphylaxis are available in the literature [6, 12, 13], involving spontaneous rupture, traumatic rupture, and iatrogenic rupture. A rare case of anaphylaxis from intravascular rupture without intraperitoneal contamination was reported in the UK in 2010 [14]. One literature review of 68 patients identified one death from anaphylaxis after traumatic rupture (1.4% mortality) [6].

The prognosis for uncomplicated hydatid liver cysts is generally favourable; however, the risk of complication is always present. Complications of cystic disease are commonly fatal, thus attempted curative treatment is always indicated.

## 4. Conclusion

Although rare, intraperitoneal rupture of hepatic (or indeed non-hepatic) cysts is a life-threatening complication of Cystic Echinococcosis and should be included in the differential diagnosis of patients in endemic areas presenting with an acute abdomen. A long history of right upper quadrant or epigastric pain prior to presentation may also suggest the diagnosis. In other parts of the world, suspicion should be raised in such presentations from patients originating from endemic areas. Anaphylaxis or severe allergic reaction may be the only presenting feature in ruptured cysts, especially after trauma, and should again raise suspicion in patients from endemic areas, especially if no other cause is evident.

Cases of intraperitoneal rupture with anaphylaxis seem to be most commonly reported by Turkish institutions; from our literature search there are no such reported cases from the UK. Vigilance in UK institutions is important given the recent influx of economic migrants from endemic areas including Eastern Europe, and the possibility of Turkey entering the EU.
